# Novel Transthyretin Amyloid Fibril Formation Inhibitors: Synthesis, Biological Evaluation, and X-Ray Structural Analysis

**DOI:** 10.1371/journal.pone.0006290

**Published:** 2009-07-21

**Authors:** Satheesh K. Palaninathan, Nilofar N. Mohamedmohaideen, Elisabetta Orlandini, Gabriella Ortore, Susanna Nencetti, Annalina Lapucci, Armando Rossello, Joel S. Freundlich, James C. Sacchettini

**Affiliations:** 1 Department of Biochemistry and Biophysics, Texas A&M University, College Station, Texas, United States of America; 2 Dipartimento di Scienze Farmaceutiche, Università Pisa, Pisa, Italy; Griffith University, Australia

## Abstract

Transthyretin (TTR) is one of thirty non-homologous proteins whose misfolding, dissociation, aggregation, and deposition is linked to human amyloid diseases. Previous studies have identified that TTR amyloidogenesis can be inhibited through stabilization of the native tetramer state by small molecule binding to the thyroid hormone sites of TTR. We have evaluated a new series of β-aminoxypropionic acids (compounds **5–21**), with a single aromatic moiety (aryl or fluorenyl) linked through a flexible oxime tether to a carboxylic acid. These compounds are structurally distinct from the native ligand thyroxine and typical halogenated biaryl NSAID-like inhibitors to avoid off-target hormonal or anti-inflammatory activity. Based on an *in vitro* fibril formation assay, five of these compounds showed significant inhibition of TTR amyloidogenesis, with two fluorenyl compounds displaying inhibitor efficacy comparable to the well-known TTR inhibitor diflunisal. Fluorenyl **15** is the most potent compound in this series and importantly does not show off-target anti-inflammatory activity. Crystal structures of the TTR∶inhibitor complexes, in agreement with molecular docking studies, revealed that the aromatic moiety, linked to the sp^2^-hybridized oxime carbon, specifically directed the ligand in either a forward or reverse binding mode. Compared to the aryl family members, the bulkier fluorenyl analogs achieved more extensive interactions with the binding pockets of TTR and demonstrated better inhibitory activity in the fibril formation assay. Preliminary optimization efforts are described that focused on replacement of the C-terminal acid in both the aryl and fluorenyl series (compounds **22–32**). The compounds presented here constitute a new class of TTR inhibitors that may hold promise in treating amyloid diseases associated with TTR misfolding.

## Introduction

Transthyretin (TTR) is a homotetrameric protein, consisting of four 127-amino acid β-sheet-rich subunits [1], and is present in mammals, birds, and reptiles [2]. Human TTR is involved in the transport of thyroxine (T4) in the cerebrospinal fluid and is a secondary carrier of T4 in plasma; approximately half of the TTR tetramer population in plasma is bound to retinol binding protein (RBP) [1], [3], [4], [5], [6], [7], [8]. TTR normally circulates as an innocuous soluble protein, but in some individuals it polymerizes to form amyloid fibrils. The fibrils are formed through a mechanism which most likely consists of a preliminary misfolding of the TTR tetramer [9], [10], [11], followed by self-assembly into amyloid fibrils [12], [13]. The result is the formation of insoluble toxic fibrillar deposits associated with many diseases. Four types of amyloidosis have been observed: senile systemic amyloidogenesis (SSA) [14], [15], familial amyloid cardiomyopathy (FAC) [15], familial amyloid polyneuropathy (FAP) [16], and central nervous system-selective amyloidosis (CNSA) [17], [18]. SSA results from the fibrillization of wild-type TTR fibril in elderly individuals [14], [15], whereas the origins of the familial diseases (FAC, FAP, and CNSA) are thought to be rooted in the fibrillogenesis of TTR mutants found in diverse populations all over the world [19]. In familial diseases, amyloid fibril aggregation may principally determine serious pathologies, including systemic and central neuropathies and cardiomyopathies leading to severe, life-threatening conditions [20].

**Figure 1:**
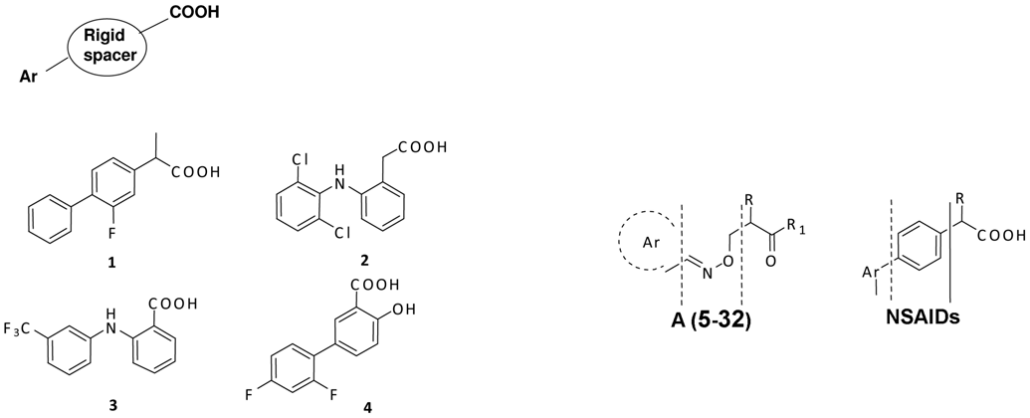
(left) General structure of NSAID inhibitors of TTR amyloidosis (1–4) and schematic representation of their common pharmacophoric portions. (Right) The two different types of spacer between the pharmacophoric portions present in synthesized compounds 5–32 of Table 1 and Table 2 with general formula A and classical NSAIDs with aryl–propionic structure, respectively.

TTR related amyloidogenesis lacks an effective therapy, although it has been observed [21] that amyloid fibril formation is prevented by the binding of the small molecule T4. Thus, stabilization by T4 analogs may underline a possible therapeutic strategy. However, the hormonal activities of T4 and its close analogs represent a safety concern. Previous reports in the literature have disclosed several small molecule families, typically sharing the halogenated biaryl motif, which stabilize the TTR tetramer [8], [22], [23], [24], [25]. These families include several nonsteroidal anti-inflammatory drugs (NSAIDs) with an arylpropionic, acetic or benzoic acid moiety (Figure 1), such as flurbiprofen (**1** or **FLP**) [22], diclofenac (**2**) [24], flufenamic acid (**FLU**) (**3**) [22], and diflunisal (**4**) [25], [26] which significantly inhibit TTR fibril formation.

**Figure 2:**
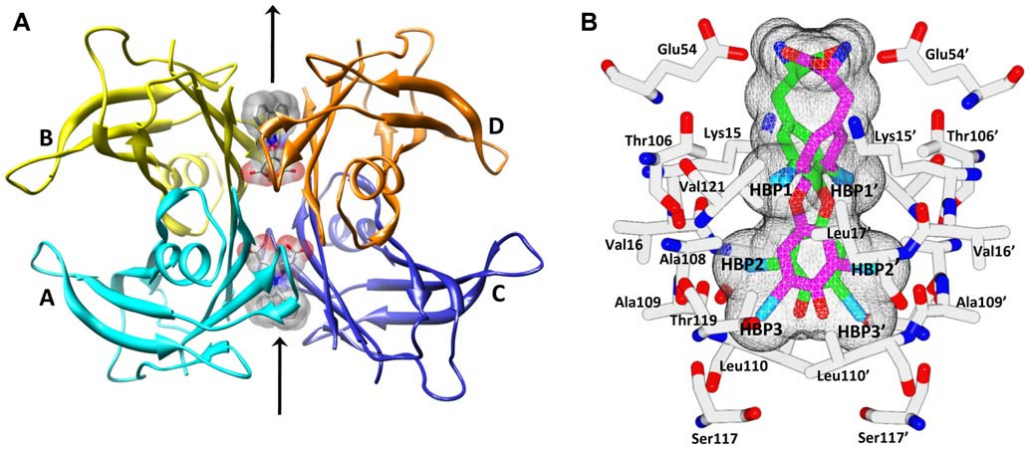
The T4 hormone binding channel of TTR tetramer. (a) Ribbon diagram of tetrameric structure of TTR with bound compound 15 (see results and discussion section). Each subunit (labeled A, B, C and D) of the tetramer is shown with its secondary structural elements and colored differently. The binding of 15 in both T4 binding pockets of TTR is shown as a stick model inside a transparent surface. The crystallographic asymmetric unit contains subunits A and B while subunits C and D were formed through crystallographic symmetry. Because of the two-fold axis along the binding channel (indicated by the arrows) a second symmetry-related binding conformation is present for the inhibitor molecule in both hormone binding sites. (b) Definition of the halogen binding pockets (HBPs) of the T4 hormone binding site of TTR based on the previously published crystal structure of T4 bound to the protein (PDB 1ROX) [4]. T4 is shown in both of its symmetry-related binding modes (shown in magenta and green inside the molecular surface mesh) that are related by a two-fold rotation axis. The carboxyl tail of T4 is positioned at the entry port of the binding site (outer binding pocket) and the iodines occupy the HBP1, HBP2, and HBP3 pockets. The inner binding pocket, HBP3, is located between the side chains of Ser117, Thr119, Ala108, and Leu110, the central HBP2 pocket is formed by the side chains of Leu17, Ala108, Ala109 and Leu110 and the outer pocket HBP1 is located between the side chains of Lys15, Leu17, Thr106 and Val121.

X-ray crystallographic studies have provided a rationale for the stabilization of the native state of TTR by T4 hormone, while offering insights into novel inhibitor designs [4], [8], [22], [27]. Previous reports of the TTR tetramer structure depicted two funnel-shaped binding sites in the T4 hormone, each defined by its dimer–dimer interface [4], [22]. Figure 2a depicts the tetrameric TTR and Figure 2b shows the close-up view of the hormone binding pocket. A junction of four Ser117 side chains may be observed, situated at the interface between the two identical T4 binding sites. Each hormone binding site can be divided into an inner and outer binding cavity. Six halogen binding pockets (HBP1, HBP1', HBP2, HBP2', HBP3 and HBP3') were also defined within each hormone binding pocket based on the positions of the halogen atoms of T4 in the TTR∶T4 crystal structure, Figure 2b [4], [22]. The inner binding cavity comprises HBP3 and HBP3', formed by the side chains of Ser117, Leu110, Thr119 and Ala108 of both subunits. The Ser117 hydroxyl groups mediate hydrogen bond interactions with bound inhibitors, as detailed in previously reported TTR∶inhibitor complex structures [8], [22], [28]. The outer binding site is composed of HBP1 and HBP1', formed primarily by residues Lys15, Leu17, Thr106, and Val121 of both subunits. HBP2 and HBP2' are positioned at the interface of the inner and outer binding cavities, comprising residues Leu17, Ala108, Ala109 and Leu110 of both subunits. The associated binding pocket is highly lipophilic allowing the HBP2 and HBP2' residues to interact favorably with the hydrophobic portions of inhibitors. Typically, TTR inhibitors and T4 bind in what is referred to as the forward binding mode, where anionic substituents like carboxylate are positioned in the outer binding pocket engaging in electrostatic interaction with the Lys15 ε-ammonium groups. However, the reverse binding mode, with the carboxylate oriented towards the inner binding pocket to hydrogen bond with Ser117 and Ser117', is not unusual and has also been observed previously in TTR complexed with diclofenac (a biarylamine), several diclofenac analogues, and some diflunisal analogs [24], [25], [29].

**Figure 3:**
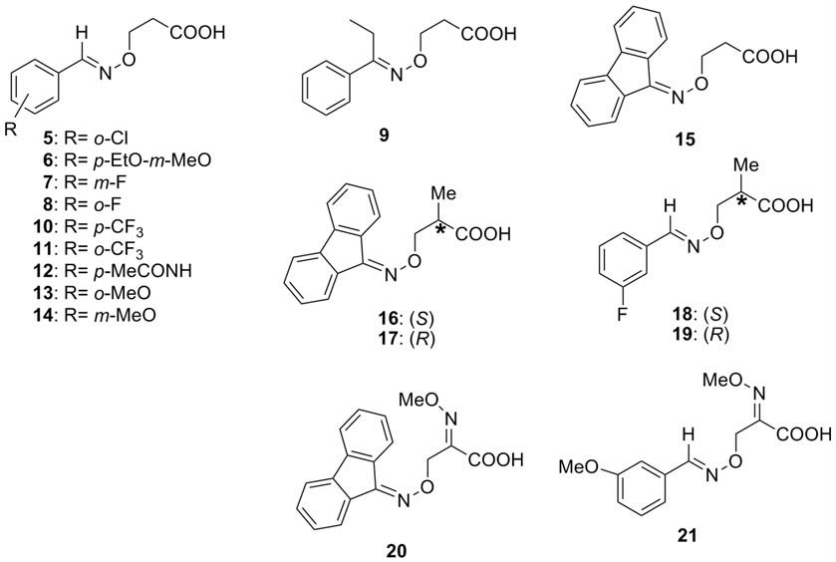
Structures of β-aminoxypropionic acid compounds evaluated in this study.

A common pharmacophore among small molecule stabilizers of the T4 hormone binding pocket of TTR tetramer is a carboxylic acid connected through a rigid spacer to an aromatic moiety (Figure 1). Based on the co-crystal structure, Zanotti et al. have shown that all-*trans*-retinoic acid, a cyclohexene-linker-acid compound, binds to TTR in a forward binding mode similar to thyroxine [30]. However, to the best of our knowledge, the aryl-linker-acid architecture has not previously been utilized for TTR tetramer stabilization. Here in this study, we have evaluated a series of previously synthesized β-aminoxypropionic acids [31], [32], [33] (compounds **5–21** in Figure 3 and Table 1), as TTR amyloid inhibitors. These compounds contain a flexible oxime-based tether between the aromatic (aryl or fluorenyl) and acidic moieties. At the outset of this investigation, we hypothesized that the greater flexibility of the oxime ether tether could allow these small molecule ligands to more fully occupy the volume of the TTR hormone binding pocket. The effect of the bulkiness of the aromatic moiety was also probed with respect to inhibitor efficacy and ligand binding mode. The diversification from the typical biaryl system was designed to yield inhibitors devoid of off-target anti-inflammatory activity, as previous TTR inhibitor designs are roughly equipotent inhibitors of both TTR [22], [34] and cyclooxygenase (prostaglandin endoperoxide synthase or COX 1–3) enzymes [35], [36], [37]. For example, **FLP** binds to TTR, COX-1 and COX-2 with similar sets of interactions and inhibits both TTR amyloidosis (70% inhibition at 7.2 µM), COX-1 (IC_50_ = 0.41 µM) and COX-2 (IC_50_ = 4.2 µM) enzyme activity [22], [36]. Based on the TTR∶**FLP** complex crystal structure, we have hypothesized that the bulkier compounds may bind specifically to TTR without inhibiting COX-1, characterized by comparatively smaller hydrophobic binding pockets [22]. Specifically, we suggested that the bulkier substituents projecting into the HBP1 and HBP2 regions of the TTR∶**FLP** pocket might significantly improve TTR-inhibitor interactions and reduce its binding affinity for COX-1 and COX-2 due to steric hindrance. In fact, replacement of the phenyl ring in the NSAIDs biaryl system with a bulkier carborane moiety greatly decreased their COX activity with the significant inhibitor efficacy of TTR dissociation [34], [38].

**Table 1:** *In vitro* acid-mediated wt-TTR (3.6 µM) amyloidogenesis inhibition activity of compounds **5–21** (see Figure 3).

Compound	% inhibition ( 3.6 µM inhibitor)
**diflunisal (4)**	63±5
** 5**	27±1.1
** 6**	12±0.51
** 7**	19±0.89
** 8**	17±2.8
** 9**	16±0.030
** 10**	13±0.30
** 11**	43±0.63
** 12**	0±0.37
** 13**	51±2.4
** 14**	17±2.1
** 15**	69±6.5
** 16**	69±3.2
** 17**	0±0.51
** 18**	27±1.5
** 19**	23±0.41
** 20**	43±0.04
** 21**	36±0.62

The percentage fibril formation was assessed by turbidity measurements at 400 nm, pH 4.4. TTR amyloidogenesis in the absence of inhibitor was assigned to be 100%. The inhibition by diflunisal [25] is included as a reference from the previously published work. For each compound, the inhibition is reported as the mean±standard error, from three independent determinations.

**Figure 4:**
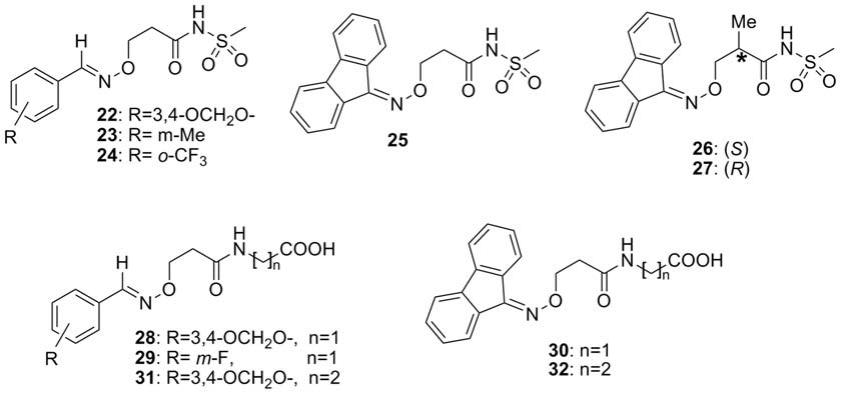
Structures of β-aminoxymethylsulfonylpropionamides (compounds 22–27), (β-aminoxypropanamido) acetic acids (compounds 28–30), and (β-aminoxypropanamido) propanoic acids (compounds 31 and 32) evaluated in this study.

An *in vitro* TTR amyloid fibril formation assay was utilized to evaluate compound efficacy and the three-dimensional crystal structures of TTR in complex with four inhibitors were solved. Molecular docking was harnessed in concert with X-ray structural studies to examine the relationship between inhibitor chemical structure, efficacy, and binding mode. In an attempt to initiate the further optimization of these TTR inhibitors, novel compounds with longer chain substitutions and enhanced flexibility were prepared (Figure 4 and Table 2): β-aminoxymethylsulfonylpropionamides (compounds **22–27**), (β-aminoxypropanamido) acetic acids (compounds **28–30**), and (β-aminoxypropanamido) propanoic acids (compounds **31** and **32**). Based on the *in vitro* assay, three of these compounds exhibited significant inhibition against TTR amyloid fibrils providing a basis for the further exploration of SAR.

**Table 2:** *In vitro* acid-mediated wt-TTR (3.6 µM) amyloidogenesis inhibition activity of compounds **22–32** (see figure 4).

Compound	% inhibition ( 3.6 µM inhibitor)
**Diflunisal (4)**	63±5
** 22**	0±5.8
** 23**	17±0.37
** 24**	56±1.2
** 25**	0±4.6
** 26**	65±2.3
** 27**	59±3.5
** 28**	2±3.8
** 29**	0±0.99
** 30**	15±0.99
** 31**	10±1.3
** 32**	10±6.5

The percentage fibril formation was assessed by turbidity measurements at 400 nm, pH 4.4. TTR amyloidogenesis in the absence of inhibitor was assigned to be 100%. The inhibition by diflunisal [25] is included as a positive control. For each compound, the inhibition is reported as the mean±standard error, from three independent determinations.

## Results

### Design and Synthesis of Aryl and Fluorenyl Families

A common pharmacophore among small molecule stabilizers (including **1–4**) of the TTR amyloidosis is a carboxylic acid connected through a rigid spacer to an aromatic moiety (Figure 1). Based on these potent TTR inhibitors, the new series of compounds (**5–21** in Figure 3 and **22–32** in Figure 4) were chosen for the current investigation. The rationale for these compounds involves two novel variations on the structures of previously known inhibitors: 1) the aryl moiety, displaying the carboxylate group, of the typical biaryl inhibitors is replaced by the C = N-O-C type linker and 2) the other ring structure in the biaryl scaffold is examined as either an aryl or fluorenyl moiety. It is noteworthy that the bulkier fluorenyl group has not been explored previously in this context.

The β-aminoxypropionic acids **5–21** were synthesized as previously reported in the literature [31], [32], [33]. The β-aminoxy-N-(methylsulfonyl)propionamides (**22–27**), the (β-aminoxypropanamido)acetic acids (**28–30**), and the (β-aminoxypropanamido)propanoic acids (**31** and **32**) were prepared as outlined in Figure S1 of the Supplementary material. In general, the condensation of β-aminoxypropionic acids with methanesulfonamide in the presence of dicyclohexylcarbodiimide and a catalytic amount of 4-dimethylaminopyridine in dimethylformamide afforded the desired compound, purified by crystallization. Reaction of the appropriate β-aminoxypropionic acids with the hydrochloride salt of the ethyl ester of glycine- or β-alanine in dimethylformamide in the presence of triethylamine and *O*-(benzotriazol-1-yl)-*N,N,N'*,*N'*-tetramethyluronium tetrafluoroborate gave the corresponding ethyl esters **33–35**, **36**, and **37**, respectively. The ethyl esters **33** and **37** thus obtained and subjected to alkaline hydrolysis to afford the desired acetic acids **28–30** or propanoic acids **31** and **32**.

### TTR amyloid fibril inhibition studies

Table 1 reports the percent inhibition of TTR fibrillization by an equimolar concentration of β-aminoxypropionic acid **5–21** inhibitor. It should be noted that this concentration was chosen to be that of TTR in plasma [8]. Of the 17 compounds tested, 3 compounds (**13**, **15**, **16**) showed more than 50% inhibition while **11** and **20** demonstrated 43% inhibition of TTR amyloidogenesis. The inhibitors may be divided into two different groups: compounds **5–15**, which lack a substituent in the carboxyl α-position, and compounds **16–21**, which have an α-methyl group (**16–19**) or an α-methoxyimino group (compounds **20** and **21**). Notably, fluorenyl compounds **15** and **16** displayed inhibition of TTR fibril formation comparable to the well-known TTR amyloid inhibitor diflunisal (69% for **15** and **16** Vs 63% inhibition for diflunisal [25]), while *ortho*-substituted aryl derivatives **11** and **13** were slightly less efficacious.

The biological results for the new β-aminoxy-N-(methylsulfonyl) propionamides (compounds **22–27**), the (β-aminoxy-propanamido) acetic acids (compounds **28–30**) and the (β-aminoxy-propanamido) propanoic acids (**31**, **32**) are reported in Table 2. Three out of 11 compounds (**24**, **26**, and **27**) showed significant inhibitory activity. Again, a fluorenyl family member, compound **26**, displayed the most potent activity (65%), with an *ortho*-substituted analog (**24**) also exhibiting good activity.

### Time course of TTR amyloid fibril inhibition for fluorenyl compounds 15, 16, and 20

**Figure 5:**
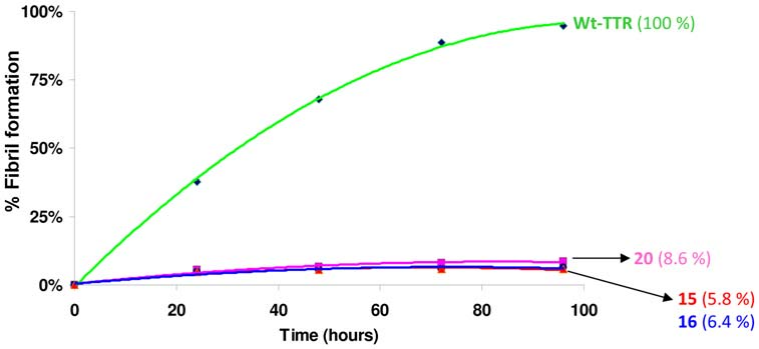
*In vitro* acid-mediated wt-TTR (3.6 µM) fibril formation in the presence of 7.2 µM inhibitors are shown plotted with wt-TTR control. Fibril formation was assessed by turbidity measurments at 0, 24, 48, 72 and 96 hours time points at 400 nm, pH 4.4. For each compound, the inhibition values are reported as the mean value (less than±5standard error), from three independent determinations.

Although the inhibitor efficacy of **15** and **16** at 3.6 µM was comparable to that of diflunisal, previous studies have shown that at 7.2 µM concentration the potency of diflunisal is increased from 63% to 96% [25]. Therefore, we evaluated **15**, **16** and **20** further at 7.2 µM concentration, monitoring the fibril formation at the 0, 24, 48, 72 and 96 hours time points (Figure 5) in three independent runs for each compound. All three inhibitors exhibited more than 90% inhibition and significantly suppressed fibril formation. Of the three tested compounds, **15** displayed the best inhibition (94%).

### Crystal structures of inhibitors 11, 13, 15 and 16 bound to TTR

**Figure 6:**
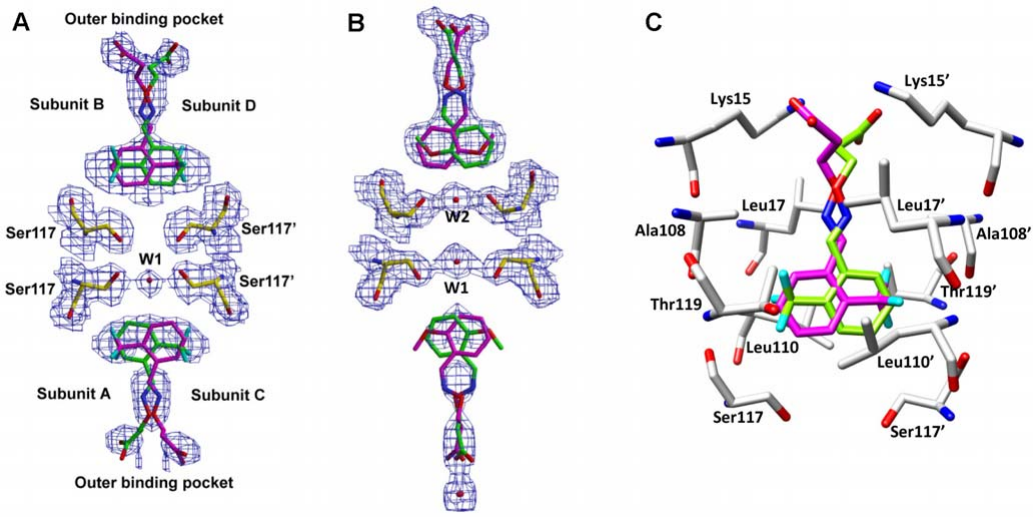
Crystal structures of inhibitors 11 and 13 bound to TTR. (a) and (b) Electron density of 11 and 13 bound with both hormone binding pockets of wt-TTR. Electron density of Shake&wARP [50] omit maps are contoured at the 1 σ level. The blob feature in XtalView [49] was applied to limit the electron density display to within 1.5 Å of the inhibitor and the final figure was rendered with Raster3D [51]. The inhibitor molecules were omitted from the model before the map calculation. (c) The binding interactions of 11 with the hormone binding pocket of TTR, the better ordered BD binding pocket is shown here. Like most of the TTR bound ligands, 11 also binds in two symmetry-related binding modes (shown in magenta and green). The key interacting residues are labeled, primed and unprimed residues refer to two neighboring symmetry related monomers comprising the T4 site. Compound 11 binds in the forward binding mode by orienting its carboxylate substituent to the outer binding pocket residue Lys15. The aryl moiety of 11 is anchored by its trifluro group to HBP3 and HBP3' of the inner most binding pocket. Like compound 11, compound 13 also binds in the forward binding mode with similar interactions (not shown here).

**Figure 7:**
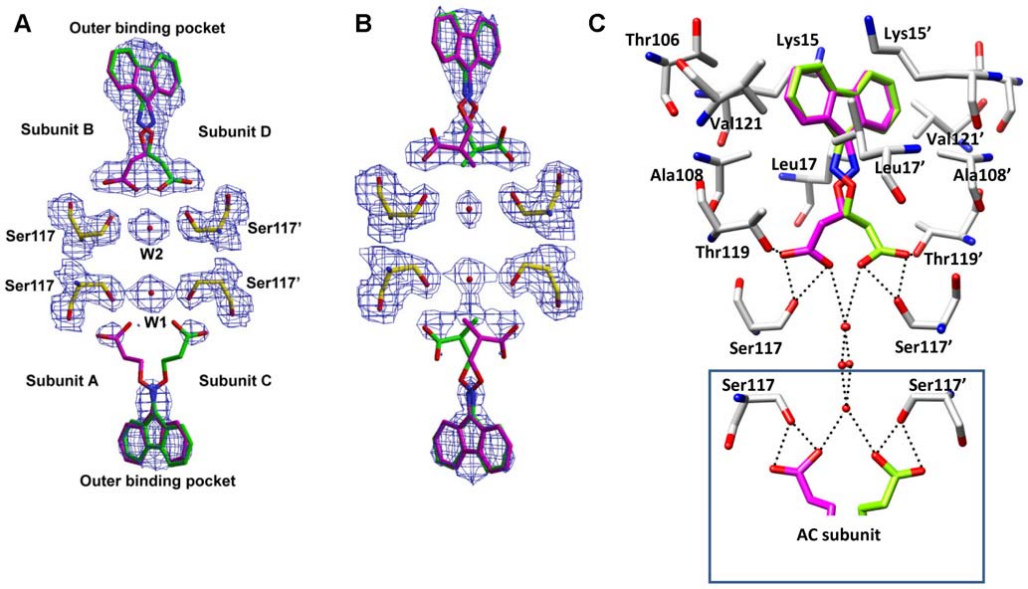
Crystal structures of inhibitors 15 and 16 bound to TTR. (a) and (b) Electron density of 15 and 16 bound with both hormone binding pockets of wt-TTR. Electron density of Shake&wARP [50] omit maps were contoured at the 1 σ level. The blob feature in XtalView has been applied to limit the electron density display to within 1.5 Å of the inhibitor and the final figure was rendered with Raster3D [51]. The inhibitor molecules were omitted from the model before the map calculation. (c) The binding interactions of 15 with the hormone binding pocket of TTR, the better ordered BD binding pocket is shown here. Inhibitor 15 also binds in two symmetry-related binding modes (shown in magenta and green). The key interacting residues are labeled, primed and unprimed residues refer to two neighboring symmetry related monomers comprising the T4 site. Compound 15 binds in the reverse binding mode by orienting its carboxylate substituent to the inner binding pocket and its fluroneyl ring to the outer binding pocket. The fluroneyl ring is optimally sandwiched between the side chain atoms of the outer binding pocket, while the corboxylate group makes a network of direct and water mediated hydrogen bonds. Leu110 and Leu110' stack on top of the linker region but are not shown for clarity. Compound 16 also binds in similar fashion (not shown here).

High-resolution X-ray crystal structures of **11**, **13**, **15** and **16** bound to wt-TTR were obtained by soaking TTR crystals with a fivefold molar excess of inhibitor for 3–4 weeks. Compounds **11** and **13** were selected for crystal structure investigation as they showed significant inhibitory activity and represent the aryl family, while the two most potent inhibitors in this report (compounds **15** and **16**) are members of the fluorenyl class. In each case, the crystals diffracted to approximately 1.8 Å resolution and the crystal data and refinement statistics for each complex structure are reported in Table 3. The overall molecular structure of the TTR∶inhibitor complexes are very similar to that of native TTR. Figure 2a shows the binding of **15** to both hormone binding pockets of the TTR tetramer. There are two hormone binding sites per tetramer (AC between the subunits A and C, and BD between the subunits B and D), each of which has an intrinsic two-fold symmetry as these are located on the crystallographic two-fold axis (indicated by an arrow in Figure 2a). All TTR∶inhibitor complex structures showed significant additional electron density in both hormone binding pockets, confirming binding of the inhibitor. The electron density corresponding to the inhibitor observed in the inner and outer hormone binding pockets of the BD dimer is well ordered in the crystal structures with compounds **11**, **13**, **15**, and **16**. (Figures 6 and 7). In contrast, the electron density of the carboxyl substituted region of these inhibitors is only partially ordered in the AC dimer (Figures 6a, 6b, 7a and 7b). The observed electron density for the substituted region and the clear electron density for the side chain atoms of the protomer allowed us to finalize the binding modes in both the AC and BD dimers without ambiguity. Moreover, similar electron density differences between the two hormone binding pockets have been observed in many of the previously reported structures including the TTR∶oxime-ether crystal structure [39].

**Table 3:** X-ray Crystallographic Data for inhibitor bound TTR structures.

	TTR∶11 (PDB^d^ ID: 3GLZ)	TTR∶13 (PDB ID: 3GS7)	TTR∶15 (PDB ID: 3GS4)	TTR∶16 (PDB ID: 3GS0)
**Data collection**				
Unit cell (Å)	42.18, 84.94, 63.42	42.17, 84.76, 63.81	43.18, 85.73, 64.77	43.39, 85.98, 65.08
Space group	P2_1_2_1_2	P2_1_2_1_2	P2_1_2_1_2	P2_1_2_1_2
Number of molecules in ASU (Z)	2	2	2	2
Resolution limit (Å)	63.37-1.8	63.76-1.8	64.82-1.8	86.07-1.9
Completeness (%)	99.5(98.9)	99.4 (98.2)	99.6 (98.9 )	98.7 (97.7)
I/σI^a^	39.5 (8.5)	16.5 (3.0)	24.7 (6.9)	14.4 (3.4)
*R*_sym_ b (%)	2.8 (16.8)	5.2 (32)	3.8(19.0)	9.0 (56.8)
**Structure Refinement**				
Resolution limit (Å)	63.37-1.8	63.76-1.8	64.82-1.8	86.07-1.9
Completeness (%)	99.5(98.9)	99.4 (98.2)	99.6 (98.9 )	98.7 (97.7)
*R*_cryst_ c (%)	22.4	22.4	22.5	22.9
*R*_free_ c (%)	27.3	26.6	27.8	26.9
Number of protomer residues in ASU	220	225	223	224
Disordered residues	A1–A9, A125–A127, B1–B9, B37–B38, B101–B103, B124–B127	A1–A9, A125–A127, B1–B9, B124–B127	A1–A9, A125–A127, B1–B9, B100–B102, B125–B127	A1–A9, A125–A127, B1–B9, B101–B102, B125–B127
Average B factor (Å^2^)	31.6	40.2	32.8	38.8
**RMS deviations from the ideal values**				
Bond lengths (Å)	0.016	0.019	0.014	0.020
Bond angles (°)	1.8	1.9	1.8	2.2
**Ramachandran statistics** (PROCHECK[52])				
Most favored (%)	92.7	89.9	91.9	89.2
Allowed (%)	7.3	10.1	8.1	10.8

Values in parentheses are for high resolution shells.

a I/sig = the mean I/sig for the unique reflections in the output file.

b *R*_sym_ = ∑_h_∑_i_ | I_hi_−〈I_h_〉|/∑_h_∑_i_ I_hi_, where I_hi_ is the *i*th observation of the reflection h, whereas 〈I_h_〉 is the means intensity of reflection h.

c *R*_cryst_ = ∑|Fo|−|Fc|/|Fo|. *R*_free_ was calculated with a fraction (5%) of randomly selected reflections excluded from refinement.

d Protein Data Bank (www.rcsb.org).

### Binding of 11 to wt-TTR

Compound **11** binds to TTR in the forward binding mode, with the carboxylate-substituted hydrophilic chain oriented in the outer binding pocket to interact with Lys15 and Lys15' (Figures 6a and 6c). In each hormone binding site, two different binding conformations of the ligand were identified with approximately equal occupancy (shown in magenta and green in Figures 6a and 6c). In the inner binding pocket of the BD dimer, the aryl ring is stacked between the hydrophobic side chains of Ala108, Ala108', Leu110, Leu110', Thr119 (through C^γ^), and Thr119' (also through C^γ^). The trifluoromethyl substituent occupies halogen binding pocket 3 (HBP3) in the inner cavity. One of the fluorine atoms of the trifluoromethyl substituent makes what may be termed as a favorable through space electrostatic interaction [40], [41] with the backbone amide N-H of Leu110 and Leu110' at a distance of 2.9 Å. In both the AC and BD dimers, Ser117 and Ser117' orient their hydroxyl groups away from the inhibitor, contributing to additional hydrophobic interactions with the aryl moiety of the inhibitor via their β-CH_2_ groups (the C6 carbon atom of the aryl moiety of **11** is at 3.3 Å distance from C^β^ carbon atom of Ser117). An ordered water molecule (labeled W1 in Figure 6a) is located between the adjacent Ser117 residues of the AC dimer facilitating hydrogen bonding interactions between the serine O^γ^ atoms (Ser117 O^γ^-W1-Ser117' O^γ^ at a distance of 2.8 Å) and is not involved in any direct interactions with the inhibitor. However, the side chain of Thr119 and Thr119' of the AC dimer makes adjustments by adopting two conformations and contributes to additional hydrophobic and hydrogen bond interactions (O^γ^ atom is at 2.9 Å and C^γ^ atom is at 3.3 Å from the closest fluorine atom of **11**) with the inhibitors. The multiple conformations of Thr119 are not observed in the BD dimer, but all other interactions of the inner binding pocket are very similar in both the AC and BD dimers. The electron density of the linker atoms is well ordered in the BD dimer and only partially ordered in the AC dimer. The electron density of the carboxylate group of the inhibitor is clearly visible. In both dimers, the linkers are stabilized by the side chains of residues Leu17, Leu17' (the closest distance from the inhibitor is 3.3 Å), Ala108, Ala108' (at 3.9 Å distance). However, in both inhibitor binding pockets, the linker oxygen and nitrogen atoms do not appear to make hydrogen bond interactions with TTR. The electron density map of all binding pocket residues was clear and well ordered. However, Lys15 and Lys15' of the AC and BD dimers showed weak electron density, indicating their flexibility, with their best fit conformations placing the N^ε^ atom at hydrogen bonding distance (3.0 Å) from the carboxyl substituent of the inhibitor.

### Binding of 13 to wt-TTR

Substitution of a methoxy group in place of the trifluoromethyl in compound **11** does not significantly affect the binding mode of **13** to TTR, while affording a slightly more potent inhibitor. Compound **13** binds to TTR in the forward binding mode (Figure 6b) with the carboxyl-substituted hydrophilic chain oriented in the outer binding pocket towards residues Lys15 and Lys15'. The electron densities of the aryl ring and linker atoms of the inhibitor are well ordered in the BD dimer and only partially ordered in the AC dimer. Thus, the binding interactions are described for the BD dimer bound inhibitor. In both T4 hormone binding pockets, the inhibitor adopts two conformations with equal occupancy (shown in magenta and green in Figure 6b). In both binding conformations, the methoxy substituent at the *ortho*- position of the aryl ring of **13** protrudes into halogen binding pockets 3 and 3′. The oxygen atom of the *ortho*-O-methyl group of **13** forms a hydrogen bond with the O^γ^ atom of Ser117 at a distance of 3.0 Å (Ser117' for the second inhibitor conformation) and the carbon atom is oriented away from Ser117 (Ser117') into HBP3 or HBP3'. In HBP3 and 3′, the methoxy group of the inhibitor makes additional hydrophobic interactions with subunit residues: Ala108 (C^β^ to O*C*H_3_ is 3.7 Å) and Thr119 (C^γ^ to O*C*H_3_ is 3.9 Å). As with the structure of bound compound **11**, the hydrophobic side chains of the inner binding pocket residues of the BD dimer (Ala108, Ala108', Leu110, Leu110', Thr119 and Thr119') stack with the aryl ring of the inhibitor. Interestingly, in contrast to **11**, water molecules (labeled W1 and W2 in Figure 6b) are located between the Ser117 and Ser117' residues of the AC and BD dimers facilitating a hydrogen bonding network through the O^γ^ atom of Ser117 residues of all subunits. Ser117 and Ser117' are also hydrogen bonded to the inhibitor. It should be noted that the oxygen atom of the methoxy group of **13** does not appear to be close enough to form a hydrogen bond (3.6 Å distance) with either water molecule. In both dimers, the linkers are stabilized by the side chains of residues Leu17, Leu17' (the closest distance from the β carbon atom of the inhibitor is 3.5 Å), Ala108, and Ala108' (3.8 Å distance from the nitrogen atom of the inhibitor). The electron density map of all binding pocket residues was clear and well ordered with the exception of the Lys15 and Lys15' in both the AC and BD dimers. With the best fit conformation, the Lys15 and Lys15' placed their N^ε^ atom at hydrogen bonding distance (2.9 Å) from the carboxyl substituent of the inhibitor. Like the TTR∶**11** structure, Thr119 of the AC dimer maintains two conformations in the inner binding pocket and interacts with the aryl ring of the inhibitor.

### Binding of 15 to wt-TTR

Inhibitor **15** binds in the reverse binding mode by orienting its carboxyl substituent towards the inner binding pocket (Figures 7a and 7c) to interact with HBP3 and HBP3' near Ser117-Ser117'. The linker region of the inhibitor is only partially ordered in AC dimer while the electron density corresponding to the BD dimer inhibitor is well ordered as shown in Figure 7a. However, in both hormone binding pockets the carboxyl group and fluorenyl moiety occupy similar positions in both inner and outer binding pockets to make comparable interactions with the residues near Ser117 and Lys15 (Figures 7a and 7c). The fluorenyl ring moiety of the inhibitor is located at the outer binding pocket formed by residues Ala108, Lys15, Leu17, Thr106, and Val121 of both TTR subunits. In contrast to the TTR∶**11** structure, all protomer residues of the binding pocket, including Lys15, were well ordered in the electron density map. Compared to the aryl compounds, the bulky fluorenyl ring occupies the outer binding pocket more extensively and forms several non-bonding interactions with the protomer residues. The fluorenyl moiety of the inhibitor is comfortably sandwiched in between the hydrophobic groups of residues Lys15 (at the closest distance of 3.8 Å from the fluorenyl ring), Lys15', Leu17 (C^δ1^ at 3.3 Å), Leu17', Thr106 (C^γ^ at 3.7 Å), Thr106', Ala108 (C^β^ at 3.4 Å), Ala108', Val121 (C^γ2^ at 3.3 Å) and Val121'. In the dimer binding pocket, the fluorenyl ring is nearly centered on the two-fold symmetry axis, giving the appearance of a single binding conformation. However, the spacer maintains two conformations (shown in magenta and green in Figures 7a and 7c) and simultaneously interacts with Ser117 and Thr119 residues of both HBP3 and HBP3' of the inner binding pocket as detailed below. In the inner binding pocket the carboxyl group forms four hydrogen bonds per subunit. Both oxygen atoms of the carboxyl group of **15** form hydrogen bonds with the side chain O^γ^ of Ser117 at distances of 2.7 and 2.8 Å. Thr119 residue orients itself towards the inhibitor and hydrogen bonds (O^γ^ of Thr119 is at 2.8 Å distance from one of the carboxylate oxygen atoms) with one of the oxygen atoms of the inhibitor carboxylate, also positioning its C^β^ atom close to the inhibitor (the carbon alpha atom to the carboxylate group of the inhibitor is at 3.6 Å distance). Interestingly, a water molecule is located between the adjacent Ser117 and Ser117' residues at the two-fold axis and forms a hydrogen bond with the carboxylate oxygen atom (3.1 Å distance) and the O^γ^ atom of Ser117 and Ser117' (3.0 Å distance). Thus, the reverse binding mode of **15** facilitates a network of hydrogen bonds in the inner binding pocket connecting the Ser117 residues of all subunits, the nearby water molecules, and the carboxylate substituents of the inhibitor (Figures 7a and 7c). The alpha- and beta-carbon atoms of the linker are also sandwiched between the side chain atoms of Leu110 (the closest distance from the alpha carbon atom of the linker is 3.8 Å) and Leu110' to further augment the binding.

### Binding of 16 to wt-TTR

Addition of an (*S*)-methyl group in the alpha position to the carboxylic group of inhibitor **15** does not affect the binding mode of the inhibitor. Compound **16** still maintains a reverse binding mode by pointing its linker toward the inner binding pocket and placing its fluorenyl ring into the hydrophobic pocket formed by residues Ala108, Lys15, Leu17, Thr106, and Val121. As in the TTR∶**15** structure, the aliphatic linker of **16** takes on two conformations with equal occupancy to simultaneously interact with both HBP3 and HBP3' residues (Figure 7b). Although the outer binding pocket interactions are very similar for both **15** and **16**, compound **16** shows notable differences in the binding interactions at the inner binding pocket of TTR. Substitution of an *(S)*-methyl group in the alpha position to the carboxylic group kinks the carboxylic acid to the side of the inhibitor, into HBP3 or HBP3'. As a result, in both conformations of the inhibitor, the methyl substituent and the alpha- and beta-carbon atoms of the linker are sandwiched between the side chain atoms of Leu110 and Leu110'; C^δ2^ of Leu110 is at 3.3 Å from the carbon alpha atom to the carboxylic group of **16**, the (S)-methyl carbon of **16** is at 3.6 Å from C^δ2^ of Leu110, and C^β^ of Leu110 is at 4.0 Å from the beta-carbon of **16**. One of the oxygens of the carboxyl group hydrogen bonds with the hydroxyl group of Ser117 (at a distance of 2.6 Å) and O^γ^ atom of Thr119 (at a distance of 2.5 Å). The second conformation of the inhibitor is generated through crystallographic two-fold symmetry and thus makes identical interactions with the hormone binding pockets of TTR. Together, the methyl substitution contributes to additional hydrophobic interactions with the inner binding pocket of TTR. The W1 and W2 water molecules observed between the Ser117 and Ser117' residues of the TTR∶**15** structure are also conserved in TTR∶**16** (in almost identical positions). However, the carboxylate group of the inhibitors does not make any hydrogen bonds with the water. Similar to the structures with bound **11**, **13** and **15**, the electron density of the linker region is only partially visible in the AC binding pocket.

### Docking of β-aminoxypropionic acids to TTR

**Figure 8:**
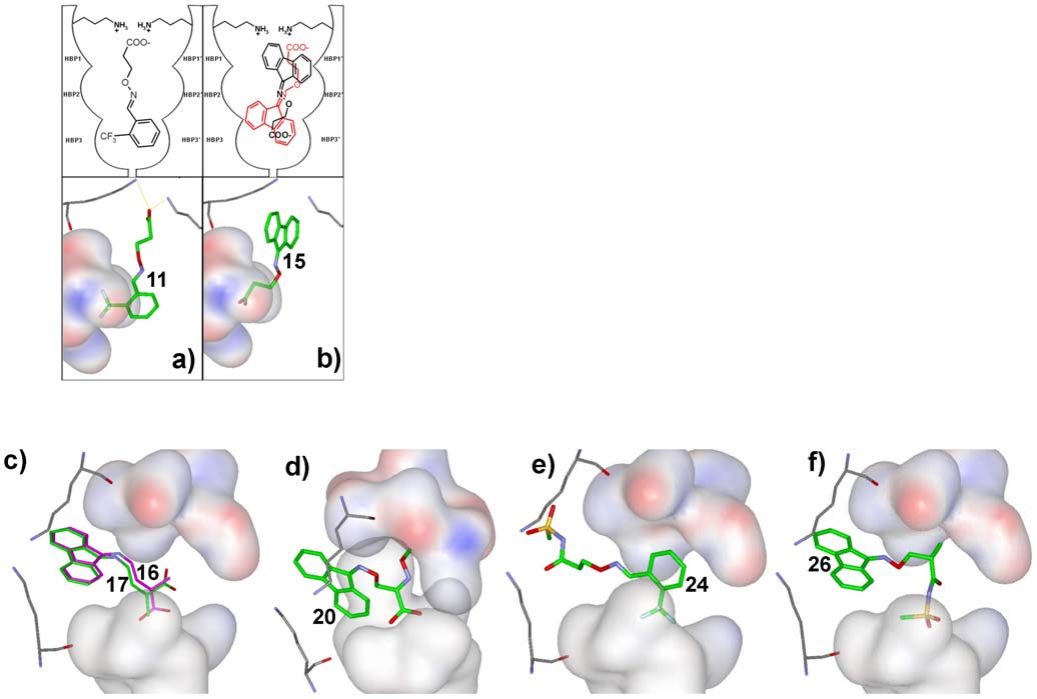
Docking of β-aminoxypropionic acids to TTR. (a) and (b) Schematic representation of compounds 11 (left) and 15 (right) in one of the TTR hormone binding sites shown with the best docking results of compounds 11 and 15, evidencing the HBP3 surface; both possible binding modes are shown for 15 (red and black). (c)–(e) Gold docking results. (c): front view of compounds 16 (magenta) and 17 (green), depicting the HBP3 surface and Lys15 of A and C monomers, (d): side view of compound 20, (e): front view of compound 24, (f): front view of compound 26.

In an effort to complement our X-ray crystallographic studies with molecular modeling exercises, we performed docking studies of compounds **5–21** with the Gold v3.1 program (Cambridge Crystallographic Data Centre), starting from the crystallographic structure of the TTR-**FLU** complex (PDB ID: 1BM7 Klabunde, 2000 #16). For the arylideneaminoxy-derivative compounds **5–14**, the Gold program predicted the typical thyroxine orientation (forward binding mode) using the Goldscore functions; The aromatic ring is inserted into HBP3, and the β-aminoxyethyl chain directs the carboxylic group towards Lys15, (see Figure 8a for compound **11**). In the docked structure, the *ortho*-CF_3_ substitution on the phenyl ring is engaged in significant non-bonding interactions with HBP3 residues Ala108, Leu110, Ser117 and Thr119. However, substituents in the *meta-* and *para-* positions are not predicted to interact with HBP3 to the same extent, since they occupy the side part of this pocket. For 9-fluorenyliden-aminoxy derivatives **15–17** and **20**, the Gold program suggested the reverse binding modes (see Figures 8b, 8c and 8d) using Goldscore functions. The TTR∶**15** and TTR∶**11** crystal structure reveals that **15** binds to the hormone binding pocket in the reverse binding mode and **11** bind in the forward binding mode with an orientation similar to the one obtained through the Goldscore algorithm. Therefore, this docking protocol was subsequently used to predict the binding of all other β-aminoxypropionic derivatives. The 9-fluorenyliden-aminoxy derivatives **16** and **17** may be placed in a reverse binding mode (Figure 8c). Only compound **16** reveals an effective interaction with HBP3. The corresponding *(R)*-enantiomer **17** inserts the methyl substituent into HBP3, and directs the carboxylic group away from the Ser117 or Ser117' residue, without any productive interactions. Interestingly, the hypothesized binding mode for compound **20** (Figure 8d) is different from the other inhibitors; the molecule interacts with HBP3 and HBP3' simultaneously, through the carboxylic moiety and the aminoxymethyl chain, respectively. Based on the docking analysis of compounds **5–21**, we have also evaluated the docking of two potent β-aminoxy-N-(methylsulfonyl) propionamides (compounds **24** and **26**) to TTR hormone binding pocket using Goldscore algorithm. As shown in Figure 8e the aryl compound **24** predicted to bind in a forward binding mode while the fluorenyl analog **26** binds in the reverse binding mode (Figure 8f) with extended interactions in HBP3s and HBP1s respectively.

## Discussion

The majority of previously reported TTR amyloid inhibitors, including diflunisal, are halogenated compounds designed to occupy one or more TTR hormone binding pockets [8]. With several potent biaryl families of TTR inhibitors available, the current drug discovery initiatives have targeted diverse chemotypes, focusing on various amyloidogenic conditions, including TTR-mutant amyloidosis, sparing off-target anti-inflammatory activity, and reducing the toxic effects of long-term NSAID administration. Recently published studies have focused on optimizing the aryl halogen substituent(s) [42], the linker between the two aryl rings [43] and minimization of cyclooxygenase (COX) inhibition (a common unwanted side effect of TTR inhibitors) [34], [38]. However, these studies were solely focused on halogenated biaryl compounds. It is interesting to note that among all compounds tested here, the most potent inhibitors (compounds **15**, **16**, **20** and **27**) are from the non-halogenated fluorenyl family, while the phenyl-substituted relatives (2 halogenated and 2 non-halogenated) showed comparatively lower inhibition. We seek to further discuss these findings, their correlation with inhibitor binding mode, and reflect on their significance with regard to known inhibitors. In particular, it is noteworthy that the fluorenyl family constitutes a novel class of TTR fibrillization inhibitors.

### Insight from aryl analogs

**Figure 9:**
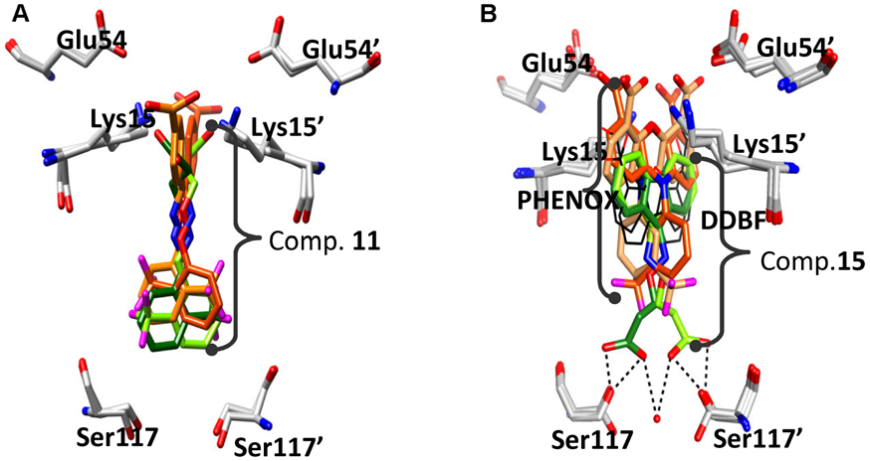
Comparison of the TTR∶11 and TTR∶15 structures with the TTR∶OE1, TTR∶DDBF, and TTR∶PHENOX structures. (a) Overlay of the binding pocket of previously published TTR∶OE1 (shown in orange red [39]) on the TTR∶11 crystal structure. Residues Ser117 and Ser117' point to the inner binding pocket while Lys15, Lys15', Glu54, and Glu54' denote the outer binding pocket of one of the T4 sites of TTR. The two binding conformations of 11 are shown in light and dark green stick format. Compared to OE1, 11 binds more deeply in the inner binding pocket and positions its carboxyl group closer to the outer binding pocket residue Lys15. (b) Overlay of the binding pocket of TTR∶DDBF [22] and TTR∶PHENOX [22] on the TTR∶15 crystal structure. Residues Ser117 and Ser117' point to the inner binding pocket while Lys15, Lys15', Glu54, and Glu54' denote the outer binding pocket of one of the T4 sites of TTR. The two binding conformations of 15 are shown in light and dark green stick format, PHENOX is shown in red and orange sticks. Both binding conformations of DDBF are shown in thin black sticks. The DDBF binds mainly to HBP1 near Lys15 while PHENOX extended its interaction from Glu54 to HBP2. The new compound 15 effectively utilizes all the HBPs located between Lys15 and Ser117. Suitable substitution in the fluorenyl ring of 15 should cover the entire binding pocket starting from Glu54 to Ser117.

The chemical structures of **11**, **13** and all other aryl derivatives discussed in Table 1 may be compared with the previously reported bisaryloxime ethers, potent inhibitors of TTR [39]. For example, 4-carboxybenzaldehyde-*O*-(2-trifluoromethylphenyl)-oxime (**OE1**) showed more than 90% inhibition at a 7.2 µM concentration against acid mediated fibrils of 3.6 µM TTR. The TTR∶**OE1** crystal structure is shown in Figure 9a [39]. This inhibitor contains an *ortho*-trifluoromethylphenyl *O*-linked through an oxime with a *para*-substituted benzoic acid and binds to TTR in the forward binding mode. Similar to the TTR∶**OE1** crystal structure, inhibitors **11** and **13** bind in the typical forward binding mode by pointing their carboxylate functionality towards the outer binding pocket residues Lys15 and Lys15' (Figure 6). In contrast to the bisaryloxime ethers, the inhibitors reported here only contain a single aryl group and the carboxyl group is directly tethered through a flexible linker. Compared to **OE1**, inhibitors **11** and **13** bind more deeply in the inner binding pocket of TTR (Figure 9a). In the TTR∶**OE1** structure, the halogenated phenyl ring moves away from the inner binding pocket and does not make significant interactions with the Ser117 and Ser117'. For inhibitors **11** and **13**, the aryl ring makes both hydrophobic and hydrogen bond interactions with Ser117, Ser117', Thr119, and Thr119' residues and points its *ortho-* substituent into the center of the HBP3 and HBP3' pockets. Taken together, the X-ray crystal structural data demonstrate that, like the **OE1** bis(aryl) family, compounds **11** and **13** can bind to TTR in the forward binding mode. However, the aryl family reported herein has the advantage of more fully occupying the inner hormone binding pockets, further stabilizing the native tetrameric structure and, in turn, inhibiting amyloid formation.

In the *in vitro* fibril formation assay, inhibitors **11** and **13** showed 43% and 51% inhibition, respectively, at a 3.6 µM concentration against an equimolar concentration of TTR. Analysis of the fibril inhibitory activity for our somewhat small set of aryl analogs suggests that TTR inhibition scales with the size of the *ortho*-substituent, with **11** (CF_3_) and **13** (OMe) having the largest groups at this position. Based on X-ray structural data and docking, the *o*-CF_3_ or *o*-OMe group is able to interact with HBP3 and HBP3' residues Ala108, Leu110, Ser117 and Thr119. When this moiety is in the *meta-* or *para-* position, these interactions with HBP3 appear to be lost, resulting in a loss of activity (cf. compounds **10** and **14** of Table 1). BBased on the crystal structures, docking studies and SAR for the aryl class, we propose that further optimization of the *ortho*-substituted aryl ring may be achieved through the direct substitution of a halogen atom at the 5-position of the aryl ring which will protrude into the HBP3' while the CF_3_ is still anchoring at HBP3 position. In addition, placement of a hydrogen bond donor and/or acceptor, i.e. hydroxyl, at the *meta*- or *para*-position may lead to enhanced interactions with Ser117/Ser117' and, thus, enhanced inhibitory activity.

Based on the inhibitor efficacy data of aryl compounds **22**, **23**, **24**, **28**, **29**, and **31** it is clear that further lengthening of the linker affects TTR fibril formation. Compounds **22**, **23**, **28**, **29**, and **31** with an aryl moiety substituted with either *meta*-Me, *meta*-F, 3,4-OCH_2_O, or the acid isostere CONHSO_2_CH_3,_ did not exhibit significant activity. However, compound **24**, featuring an *ortho*-trifluoromethyl group and the CONHSO_2_CH_3_ modification of the linker, showed a 13% improvement in fibril inhibition activity over **11**. Based on the docking analysis (Figure 8e), the *ortho*-CF_3_ group was predicted to be critical for anchoring the aryl moiety of **24** to the inner binding pocket, favoring the forward binding mode. Interestingly in the outer binding pocket, the acylsulfonamide moiety of **24** may mimic the negative charge of the carboxylate in **11**, while having the potential to make additional hydrogen bonds with Lys15 and Lys15' residues. The sulfonamide pendant methyl group may also interact with the HBP1 and HBP1' pockets of the TTR outer binding pockets. Clearly, further optimization of the terminal carboxylic acid/acid isostere, in conjunction with continued examination of the role of the *ortho-*aryl substituent, will be necessary to clarify the precise structural requirements for enhanced inhibition of fibrillization.

### Insight from the fluorenyl compounds

Compared to the aryl family, the fluorenyl derivatives presented herein do not have close analogs that have previously been well-characterized as TTR inhibitors. Dibenzofuran-4,6-dicarboxylic acid (**DDBF**) [22] and *m*-trifluoromethyl-substituted *N*-aryl phenoxazine (**PHENOX**) have been explored with the hypothesis that the bulkier tricyclic moieties should bind with higher affinity to the TTR hormone binding pocket [22]. The TTR∶**DDBF** co-crystal structure showed that the tricyclic ring of **DDBF** effectively utilizes the hydrophobicity of the outer binding pocket and binds in a forward binding mode, orienting its directly linked carboxyl moiety towards Lys15. However, the inner binding pocket is completely unoccupied (Figure 9b), and as a result **DDBF** exhibited only moderate inhibition (46%) at a 3.6 µM concentration [22]. Substituting a halogenated aryl ring for one of the benzene rings of **DDBF** increased its inhibitory activity to ∼70% [22], but the binding mode has yet to be explored [22]. In the case of the TTR∶**PHENOX** structure [22], the inhibitor binds in the outer binding pocket between Glu54 and Thr119 (Figure 9b). The biaryl system facilitated additional hydrophobic interactions with the HBP1 residues (Figure 9b) and positioned its carboxyl group close to Glu54 to form additional hydrogen bond interactions, resulting in an improved fibrillization inhibition [22] of 73% inhibition at 3.6 µM. The fluorenyl compounds presented in Table 1 constitute a new class of inhibitors as they can span the entire binding pocket and simultaneously interact with both the inner and outer binding pockets. In the TTR∶**15** and TTR∶**16** structures, the tricyclic moiety of the inhibitor completely fills the outer binding pocket and the carboxylated chain adopts two conformations to interact with both the HBP3 and the HBP3' residues of the inner binding pocket. Consequently, a water-mediated hydrogen bonding network is enabled, engaging neighboring TTR subunits AC and BD (Figure 7). The fluorenyl (outer pocket)-tether (linker space)-carboxyl (inner pocket) architecture of **15** and **16** fully occupies the space between Lys15 and Ser117 and stabilizes the tetrameric structure of TTR by tethering each of its subunits through numerous interactions. The fluorenyl moiety is sandwiched between the hydrophobic groups of residues Lys15, Lys15', Leu17, Leu17', Thr106, Thr106', Ala108, Ala108', Val121 and Val121' in the outer binding pockets of both AC and BD dimer. The carboxyl group simultaneously interacts with Ser117 and Thr119 residues of both HBP3 and HBP3' and facilitates a network of hydrogen bonds in the inner binding pocket connecting the Ser117 residues of all subunits, the nearby water molecules, and the carboxylate substituents of all the inhibitors. In addition, the linker carbon atoms are also sandwiched between the side chain atoms of Leu110 and Leu110' to further augment the inhibitor protomer interactions. Thus, **15** and **16** exhibit approximately 70% inhibitory activity at 3.6 µM concentration and more than 90% inhibitory activity at 7.2 µM against 3.6 µM TTR fibrils. It is interesting to note that the *R*-enantiomer (**17**) of the most potent inhibitor (**16**) of this series is completely inactive, suggesting that the conformation of the linker is critical for the activity of **16**. This is, however, not the case with the modified linker compounds **26** and **27**, in which the activity remained similar for both *S-* and *R*-enantiomers.

The X-ray structural data demonstrate that the aromatic group (phenyl or fluorenyl) is what determines the binding mode of the TTR inhibitor in this series. Particularly, the complementarity of the fluorenyl moiety to the outer binding pocket supports this conclusion. The two-carbon linker of **15** compliments the reverse binding of fluorenyl derivatives and yielded the most potent compound of this series. It is interesting to note that alterations to the linker, including stereodefined α-methylation (to afford **16** and **17**) and introduction of an α-methoxyimino moiety (to afford **20**), did not improve inhibition of fibrillization. Clearly, the stereochemistry of the appended α-methyl group is important and this information should be of utility in future designs. The limited number of replacements for the terminal carboxylic acid in the fluorenyl series produced mixed results. In the case of substitution of the acid with an acylsulfonamide, we observed a complete loss of activity (**15** versus **25**), maintenance of inhibition (**16** versus **26**), and significant gain of efficacy (**17** versus **27**). Clearly, a larger subset of compounds will be required to clarify this SAR along with the critical structural insights that X-ray crystallography can provide. Similar efforts may also allow us to realize benefit from the use of amide-acid termini, such as in **30** and **31**, which currently do not exhibit a benefit compared to the carboxylic acid terminated **15**. Furthermore, we will seek to examine the role of substitutions on both the fluorenyl ring and two-carbon linker of **15** to further optimize this potent class of fibrillization inhibitors.

### Concluding remarks

Previous studies have shown that diverse families of biaryl compounds possess potent inhibitory activity against TTR amyloidosis. We have evaluated a novel family of inhibitors, featuring an aryl or fluorenyl substituent tethered through a flexible oxime to a carboxylic acid, or methylsulfonamide group. Of the 28 compounds tested, based on the *in vitro* fibril formation assay, eight compounds showed significant inhibition of TTR amyloidogenesis at a concentration of 3.6 µM, equal to the concentration of tetrameric wt-TTR. The x-ray crystal structures of the TTR–inhibitor complexes presented here illustrates the key molecular features for inhibitor binding and provide the structural basis for the stabilization of the native tetrameric TTR. The crystallographic and docking studies also suggest that the aryl compounds prefer the forward binding mode and the flurorenyl analogs orient in a reverse binding mode. Analysis of the TTR∶inhibitor complexes reveals the advantage of the fluorenyl group over the aryl moiety, in combination with the flexible oxime based tether, in tetramer stabilization. With regard to further diversification from T4, none of these fluorenyl compounds, including four potent TTR amyloid inhibitors, contain halogen substitutions, in striking contrast to the majority of previously reported biaryl inhibitors. Fluorenyl compound **15** exhibited the best activity, reducing fibril formation by nearly 70% at 3.6 µM concentration and 95% at 7.2 µM concentration. Another important advantage of **15** is that it is devoid of anti-inflammatory activity in an *in vivo* carrageenan-induced paw edema assay in rats [31], [33]. This selectivity, in contrast to NSAID-based TTR inhibitors such as Flu, is hypothesized to derive from the bulky nature of the fluorenyl moiety. The bulkier fluorenyl system may render **15** incompatible with the comparatively smaller COX enzyme binding pockets (Supplementary Figure S2). Thus, **15** may represent a good starting point for a new family of compounds that stabilize the TTR tetramer, while sparing off-target anti-inflammatory activity and reducing the toxic effects of long-term NSAID administration.

## Materials and Methods

### Chemistry

Melting points were determined on a Kofler hot-stage apparatus and are uncorrected. ^1^H NMR spectra of all compounds were obtained with a Gemini 200 spectrometer operating at 200 MHz, in a ca. 2% solution of CDCl_3_. Analytical TLCs were carried out on 0.25 mm layer silica gel plates containing a fluorescent indicator; spots were detected under UV light (254 nm). Mass spectra were detected with a Hewlett Packard 5988A spectrometer. Evaporation was performed *in vacuo* (rotating evaporator); Na_2_SO_4_ was always used as the drying agent. Elemental analyses (C, H, N) were performed in our analytical laboratory and agreed with theoretical values to within±0.4%.

### General procedure for the synthesis of the propionamides 22–27

A solution of the appropriate β-aminooxy acid A (1.19 mmol) and the methanesulfonamide (1.19 mmol) in anhydrous DMF (3 mL) was stirred at r.t. under N_2_ atmosphere, in presence of DCC (1.19 mmol) and a catalytic amount of DMAP (0.119 mmol), until the disappearance of the starting carboxyl compound (TLC, 48 h). After this time the mixture was taken up with Et_2_O, cooled at 0°C for 50 minutes and the resulting solid precipitate was filtered. The Et_2_O solution was acidified with acetic acid and washed with H_2_O and brine. Evaporation of the organic layer afforded a residue, which was crystallized from appropriate solvents to give the pure desired compounds **22–27**:

#### (*E*)-N-(methylsulfonyl)-3-(3,4-methylendioxy)-(benzylideneaminooxy)-propionamide (22)

(45%) m.p 130–131°C (CHCl_3_–hexane); ^1^H NMR 2.82 (t, 2H, J = 5.2 Hz), 3.31 (s, 3H) , 4.42 (t, 2H, J = 5.2 Hz) 6.02 (s, 2H), 6.80–7.23(m,3H) , 8.04(s, 1H), 9.28(brs, 1H). C_12_H_14_N_2_O_6_S (C, H, N).

#### (*E*)-N-(methylsulfonyl)-3-(3-methyl-benzylideneaminooxy)propionamide (23)

(45%) m.p 107–108°C (hexane); ^1^HNMR 2.29 (s, 3H), 2.84 (t, 2H, J = 5.4 Hz), 3.31 (s, 3H), 4.46 (t, 2H, J = 5.4 Hz), 7.30–7.46 (m, 4H), 8.11(s, 1H), 9.10(brs, 1H). C_12_H_16_N_2_O_4_S (C, H, N).

#### (*E*)-N-(methylsulfonyl)-3-(2-trifluoromethyl-benzylideneaminooxy)-propionamide (24)

(40%) (CHCl_3_–hexane) m.p 116–118°C ^1^H NMR 2.83 (t, 2H, J = 5.4 Hz), 3.29 (s, 3H) , 4.52 (t, 2H, J = 5.4 Hz), 7.32–7.45 (m, 3H) ,7.99–8.01(m, H), 8.45 (s, 1H), 9.45(br s, 1H). C_12_H_13_F_3_N_2_O_4_S (C, H, N).

#### 3-(9-fluoren-9-ylideneaminooxy)-N-(methylsulfonyl)propionamide (25)

(20%) m.p 173–174°C (*i*-PrOH) ^1^H NMR 2.96 (t, 2H J = 5.4 Hz), 3.26 (s, 3H, ), 4.71(t, 2H, J = 5.4 Hz), 7.31–7.64 (m, 6H), 7.82(d, 1H, J = 7.6 Hz), 8.21(d, 1H, J = 7.6 Hz) 9.25(brs, 1H). C_17_H_16_N_2_O_4_S (C, H, N).

#### (S)-(26) and (R)-(27) 3-(9-fluoren-9-ylideneaminooxy)-2-methyl-N-(methylsulfonyl) propionamides

(*S*)-**26** (35%) [*α*]_D_^22^+4.2 (CHCl_3_, c = 1.18); and (*R*)- **27** (30%) [*α*]_D_^22^−4.8 (CHCl_3_, c = 1.09); m.p 182°–183°C (CH_2_Cl_2_-hexane ) ^1^H NMR 1.32(d, 3H, J = 7.0 Hz), 3.07 (m, 1H), 3.22 (s, 3H), 4.54 (m, 2H), 7.26–7.67 (m, 6H), 7.78(d, 1H, J = 7.4 Hz), 8.20(d, 1H, J = 7.4 Hz), 8.90(br s, 1H). C_18_H_18_N_2_O_4_S (C, H, N). The compounds (*S)*-**26** and (*R)*-**27** possess an enantiomeric purity >96% determined by ^1^H NMR analysis using (*R)*-cinconidine as a chiral resolving agent. An equimolar mixture of (*S*)-(**26**)- and (*R*)-(**27**)-3-(9-fluoren-9-ylideneaminooxy)-2-methyl-N-(methylsulfonyl) propionamides with *(R)*-cinconidine in CDCl_3_ displayed in its ^1^H NMR spectrum two doublets at 1.25 and 1.21 ppm, attributable to 2-methyl protons of *(R)*-**27** and (*S)*-**26** stereoisomers, respectively.

### General procedure for the synthesis of the ethyl esters 33–37

To a solution of appropriate β-aminooxy acid A (3.58 mmol) in anhydrous DMF (28.0 mL) in the presence of Et_3_N (1.5 mL) was added portionwise the glycine ethyl ester hydrochloride (3.78 mmol) or β−alanine ethyl ester hydrochloride and TBTU (O-benzotriazol-1-yl-N,N,N',N'-tetramethyluronium tetrafluoroborate, 8.4 mmol). The resulting solution was stirred at r.t. for 4 h. After this time the mixture was taken up with AcOEt and washed with H_2_O, a solution of 5% aqueous NaHCO_3_, an aqueous solution of 0.1 M HCl, and brine. Evaporation of the dried organic layer afforded a residue which was crystallized from appropriate solvents to give the pure desired compounds **33–37** :

(*E)*-ethyl 2-(3-(3,4-methylenedioxy)-benzylideneaminoxy) propanamido)acetate (**33**): (50%) m.p 90–91°C (*i-*Pr ether), ^1^H NMR 1.27 (t, 3H, J = 6.4 Hz), 2.86 (t, 2H, J = 5.4 Hz), 4.03–4.47 (m, 6H), 5.95 (s, 2H ), 6.35(br s,1H), 6.72–7.30 (m, 3H), 7.97 (s, 1H). C_15_H_18_N_2_O_6_ (C, H, N).

(*E*)-ethyl 2-(3-(4-fluoro)-benzylideneaminoxy) propanamido)acetate (**34**): (52%) m.p. 102–103°C (CHCl_3_-hexane) ^1^H NMR 1.26 (t, 3H, J = 6.4 Hz), 2.87 (t, 2H, J = 5.6 Hz), 4.03–4.47 (m, 6H), 6.35 (br s,1H), 6.09–7.51 (m, 4H) 8.04 (s, 1H). C_14_H_17_FN_2_O_4_ (C, H, N).

Ethyl 2-(3-9H-fluoren-9-ylideneaminoxy) propanamido)acetate (**35**): (71%) m.p 99–100°C (*i-*Pr ether-hexane). ^1^H NMR 1.22 (t, 3H, J = 6.4 Hz), 2.80 (t, 2H, J = 5.6 Hz), 3.97–4.26 (m, 4H), 4.67 (t, 2H, J = 5.6 Hz), 6.25 (br s,1H), 7.12–7.88 (m, 7H), 8.21 (d, 1H, J = 7.6 Hz). C_20_H_20_N_2_O_4_ (C, H, N).

(*E*)-ethyl 3-(3-(3,4-methylendioxy)-benzylideneaminoxy) propanamido) propanoate (**36**): (45%) m.p 72°–73°C (hexane), ^1^H NMR 1.23 (t, 3H, J = 6.4 Hz), 2.6 (m 4H), 3.51 (q, 2H, J = 5.6 Hz), 4.01 (q, 2H J = 6.4 Hz), 4.36 (q, 2H J = 5.6 Hz), 5.96 (s, 2H, ), 6.40 (brs,1H), 6.80–7.21 (m, 3H), 7.95 (s, 1H). C_16_H_20_N_2_O_6_ (C, H, N).

Ethyl 3-(3-9H-fluoren-9-ylideneaminoxy) propanamido) propanoate (**37**): (55%) m.p 115°–116°C (CHCl_3_-hexane), ^1^H NMR 1.14 (t, 3H, J = 6.4 Hz), 2.44 (t, 2H, J = 6.4 Hz), 2.72 (t, 2H, J = 5.6 Hz), 3.50 (q, 2H, J = 6.4 Hz), 3.95 (q, 2H J = 7.2 Hz), 4.65 (t, 2H, J = 5.6 Hz), 6.40 (br s, 1H), 7.22–7.78 (m, 7H), 8.17 (d, 1H, J = 7.6 Hz). C_21_H_22_N_2_O_4_ (C, H, N).

### General procedure for the synthesis of the acids 28–32

To a solution of ethyl esters of acetate derivatives **33–35** or the ethyl esters of propanoate derivatives **36, 37** (0.93 mmol) in EtOH/H_2_O (1∶1, 6 mL) was added a solution of solid KOH (0.025 mmol) in EtOH (8 mL). After 24 h at r.t., the solvent was evaporated and the residue was taken up with H_2_O and washed with Et_2_O. The aqueous layer was acidified to pH 4 with 10% aqueous HCl and then extracted with Et_2_O. Evaporation of washed (H_2_O) and dried organic layer gave a solid residue which was crystallized from the proper solvent to give pure **28–32**:

(*E*)-2-(3-(3,4-methylendioxy)-benzylideneaminoxy) propanamido)acetic acid (**28**): (30%) m.p. 110°–112° (hexane).^ 1^H NMR 2.68 (t, 2H, J = 5.6 Hz ), 4.0, 4.06 (d, 2H, J = 4.8 Hz), 4.39 (t, 2H, J = 5.6 Hz), 5.95 (s, 2H) 6.55 (br s,1H), 6.71–7.24 (m, 4H), 7.99 (s, 1H). C_13_H_14_N_2_O_6_ (C, H, N).

(*E*)-2-(3-(4-fluoro)-benzylideneaminoxy)propanamido)acetic acid (**29**): (45%) m.p 126–128°C (hexane).^ 1^H NMR 2.65 (t, 2H, J = 5.6 Hz), 3.99 (d, 2H, J = 4.8 Hz) 4.42 (t, 2H, J = 5.6 Hz) 6.60 (br s,1H) 6.90–7.72 (m, 4H), 8.03 (s, 1H). C_12_H_13_FN_2_O_4_ (C, H, N)

2-(3-9H-fluoren-9-ylideneamiooxy)propanamido)acetic acid (**30**): (71%) m.p 104°–105°C (*i-*Pr ether).^1^H NMR 2.79 (t, 2H, J = 5.6 Hz), 3.97 (d, 2H, J = 4.8 Hz), 4.66 (t, 2H, J = 5.6 Hz), 6.60 (br s,1H), 7.12–7.88 (m, 7H), 8.20 (d, 1H, J = 7.6 Hz). C_18_H_16_N_2_O_4_ (C, H, N).

(*E*) 3-(3-(3,4-methylendioxy) benzylideneaminoxy) propanamido)propanoic acid (**31**): (40%) m.p 85°–87°C (hexane).^1^H NMR 2.48–2.67(m, 4H), 3.51(q, 2H, J = 5.6 Hz), 4.35 (t, 2H, J = 5.6 Hz ), 5.94 (s, 2H ), 6.55 (brs,1H), 6.67–7.21 (m, 3H) 7.93 (s, 1H). C_14_H_16_N_2_O_6_ (C, H, N).

3-(3-9H-fluoren-9-ylideneamiooxy) propanamido) propanoic acid (**32**): (45%) m.p 112°–114°C (CHCl_3_, hexane). ^1^H NMR 2.40–2.79(m, 4H), 3.53(q, 2H, J = 5.6 Hz), 4.65(t, 2H, J = 5.6 Hz), 6.55 (br s,1H), 7.26–7.67 (m, 8H) . C_19_H_18_N_2_O_4_ (C, H, N).

The *E* configuration around the C = N double bond of the β-aminoxy-N-(methylsulfonyl)propionamides **22–24**, the (β-aminoxy-propanamido)-acetic acids **28**,**29** and the propanoic acid **31** was assigned on the basis of the knowledge of the configuration of starting acids and on the basis the observation that in their ^1^H NMR spectra the proton linked to the oxymic carbon atom, resonates at chemical shift values identical or very close to those of the same proton of starting acids of *E* configuration

### Molecular modeling

The crystal structure of the TTR∶**FLU** dimeric complex (PDB ID: 1BM7 [27]) was used to generate the correspondent tetramer through the Unit Cell Tool of the Chimera program [44]. All of the inhibitors were submitted to a conformational search of 1000 steps with a 2 kcal/mol energy window and then minimized using the conjugated gradient method until a convergence value of 0.05 kcal/mol by means of the MACROMODEL (*Macromodel*, ver. 8.5; Schrödinger Inc.: Portland, OR, 1999) program. The algorithm used was the Monte Carlo method with MMFFs as the force field and a distance-dependent dielectric constant of 4.0. The docking studies were carried out through the standard mode of GOLD 3.1 [45], which uses a genetic algorithm search strategy. A binding site was defined as all atoms of TTR within 5 Å of the FLU, and the Cavity Detection algorithm [46] was enabled. The number of generated poses was set to 20, without the early termination option, and the default calculation mode was selected. By default, the GA (Genetic Algorithm) run comprised 100,000 genetic operations on an initial population of 100 members divided into five subpopulations, and the annealing parameters of the fitness function were set at 3.0 for van der Waals and 2.5 for hydrogen bonding. The two fitness functions implemented in Gold, GoldScore was used to identify the better binding mode.

### Fibril formation assay

Wild-type TTR was purified from an *E. coli* expression system and the rate of acid-mediated fibril formation and inhibitor efficacy of each compound was determined by monitoring the turbidity of wt-TTR at pH 4.4, as described previously [22], [25], [27]. Each compound was dissolved in DMSO at a concentration of 720 µM starting from a primary stock solution of 7.2 mM. Five microliters of a solution of the compound being evaluated was added to 495 µL of a 7.2 µM TTR solution in 10 mM phosphate (pH 7.6), 100 mM KCl, 1 mM EDTA buffer, allowing the compound to incubate with TTR for 30 min at room temperature. Five hundred microliters of 200 mM acetate buffer (pH 4.2), 100 mM KCl, 1 mM EDTA was added to yield final TTR and inhibitor concentrations of 3.6 µM each and a final pH of 4.4. The final 1 mL mixture was vortexed, then incubated at 37°C for 72 hr, after which the tubes were vortexed, and the optical density was measured at 400 nm in a disposable UV cuvette. All assays were performed in triplicate. The percentage of fibril formation was determined by normalizing each optical density by that of TTR without inhibitor, defined to be 100% fibril formation. Control solutions of each compound in the absence of TTR were tested, all compounds were soluble and none absorbed appreciably at 400 nm, ensuring that turbidity was the result of TTR amyloid formation.

### X-ray Data collection and structure determination

Crystals of wt-TTR were obtained from 5–7 mg/mL protein solutions (in 100 mM KCl, 1 mM EDTA, 10 mM sodium phosphate, pH 7.0, 0.3 M ammonium sulfate) equilibrated against 2 M ammonium sulfate in hanging drops. All TTR∶ligand complexes were prepared from crystals soaked with a fivefold molar excess of ligand for 3–4 weeks to ensure saturation of both binding sites without affecting the diffraction quality of the crystals. An R-axis IV++ detector coupled to an Rigaku Micromax 007 rotating anode X-ray generator was used for data collection of all four complex structures. A single crystal was placed in paratone oil as a cryoprotectant and cooled to 120 K for diffraction experiments. Crystals of all TTR∶ligand complex structures are isomorphous with the apo crystal form with the space group P2_1_2_1_2 with two subunits in the asymmetric unit (Table 3). All data sets were processed and scaled with the Crystal Clear suite (Rigaku Corporation).

### Structure refinement

The protein atomic coordinates for wt-TTR from the Protein Data Bank (accession number 1BMZ [27]) were used as a starting model during the rigid body refinement in CCP4-Refmac [47], [48]. For each binding pocket of the TTR tetramer, the resulting difference Fourier maps revealed two ligand-binding conformations. The electron density corresponding to the inhibitor observed in the inner and outer hormone binding pockets of the BD dimer was well ordered in all TTR∶ligand crystal structures (Figures 6 and 7). In contrast, the electron density of the carboxyl substituted region of these inhibitors was only partially ordered in the AC dimer. However, the ligands could be unambiguously placed into the existing density of both hormone binding pockets and were included in the crystallographic refinement. The subsequent map-fitting was done in XtalView/Xfit [49] using the unbiased weighted electron density map calculated by the Shake&wARP bias removal protocol [50]. All binding conformations of the ligand were in good agreement with the unbiased annealed omit maps as well as the Shake&wARP unbiased weighted maps phased in the absence of the inhibitor. Final cycles of the refinement were carried out by the maximum likelihood restrained refinement protocol of CCP4-Refmac [47]. Due to the lack of interpretable electron densities in the final map, the nine N-terminal and three C-terminal residues were not included in the final model. A summary of the crystallographic analysis is presented in Table 3. Water molecules were identified using XtalView/Xfit. The figures were prepared using XtalView [49], Raster3D [51] and UCSF Chimera [44]. The atomic coordinates of the TTR inhibitor complexes have been deposited in the Protein Data Bank with the codes 3GLZ, 3GS7, 3GS4, and 3GS0.

## Supporting Information

Figure S1 Chemical scheme for the synthesis of compounds 22–32.
(0.05 MB PPT)

Figure S2 Side by side comparison of the binding pockets of TTR∶15 structure and the prostaglandin binding channel of COX-2 (PDB 3PGH). Left: Hormone binding channel of TTR with bound 15, the halogen binding pockets are labeled according to the manuscript. Right: Binding of the flurbiprofen (FLP) into the prostaglandin binding channel of COX-2 (PDB 3PGH). The binding channel has two entrances, one on the top and one on the left. The COOH group of flurbiprofen is positioned at one of the entries close to residue Arg120 and thus allows the formation of a salt bridge. As in the TTR∶biphenyl compounds, the protein:ligand interactions are augmented by hydrophobic interactions between the biphenyl moiety of the drug and hydrophobic protein residues. In contrast to Flu, the newly designed compounds based on bulkier 15 are less compatible with the COX enzyme binding pockets.
(0.68 MB PPT)
