# Supplementary figures and images for: Novel Transthyretin Amyloid Fibril Formation Inhibitors: Synthesis, Biological Evaluation, and X-Ray Structural Analysis
Source: PLoS One. 2009 Jul 21;4(7):e6290. doi: 10.1371/journal.pone.0006290 (PMC2709434; doi:10.1371/journal.pone.0006290)

## Slide 1
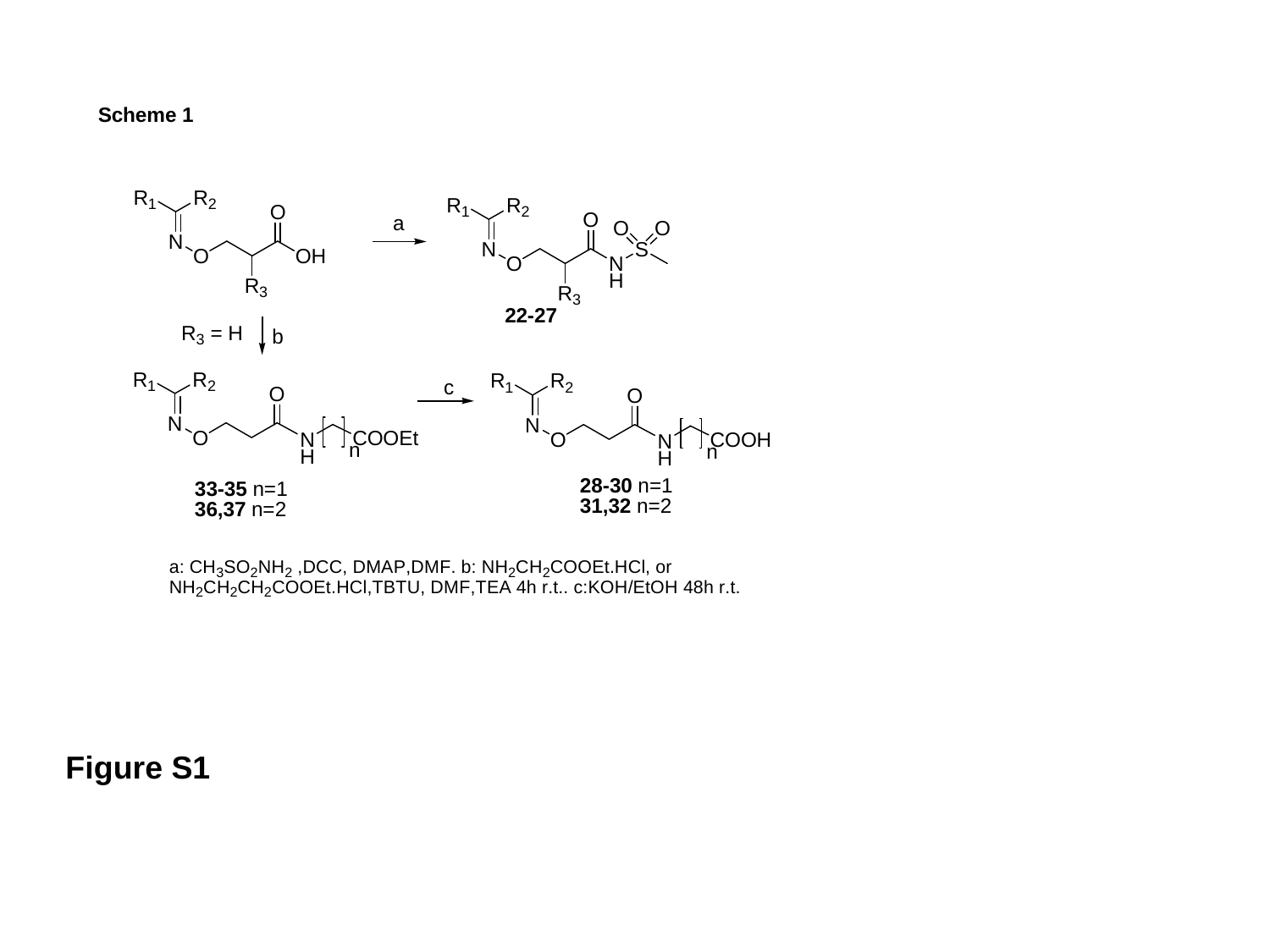

Figure S1

Supplement: Figure S1 — Chemical scheme for the synthesis of compounds 22–32. (0.05 MB PPT) [file pone.0006290.s001.ppt]
