# Supplementary material for: Novel Transthyretin Amyloid Fibril Formation Inhibitors: Synthesis, Biological Evaluation, and X-Ray Structural Analysis
Source: PLoS One. 2009 Jul 21;4(7):e6290. doi: 10.1371/journal.pone.0006290 (PMC2709434; doi:10.1371/journal.pone.0006290)
Supplement: Figure S2 — Side by side comparison of the binding pockets of TTR∶15 structure and the prostaglandin binding channel of COX-2 (PDB 3PGH). Left: Hormone binding channel of TTR with bound 15, the halogen binding pockets are labeled according to the manuscript. Right: Binding of the flurbiprofen (FLP) into the prostaglandin binding channel of COX-2 (PDB 3PGH). The binding channel has two entrances, one on the top and one on the left. The COOH group of flurbiprofen is positioned at one of the entries close to residue Arg120 and thus allows the formation of a salt bridge. As in the TTR∶biphenyl compounds, the protein:ligand interactions are augmented by hydrophobic interactions between the biphenyl moiety of the drug and hydrophobic protein residues. In contrast to Flu, the newly designed compounds based on bulkier 15 are less compatible with the COX enzyme binding pockets. (0.68 MB PPT) [file pone.0006290.s002.ppt]

## Slide 1
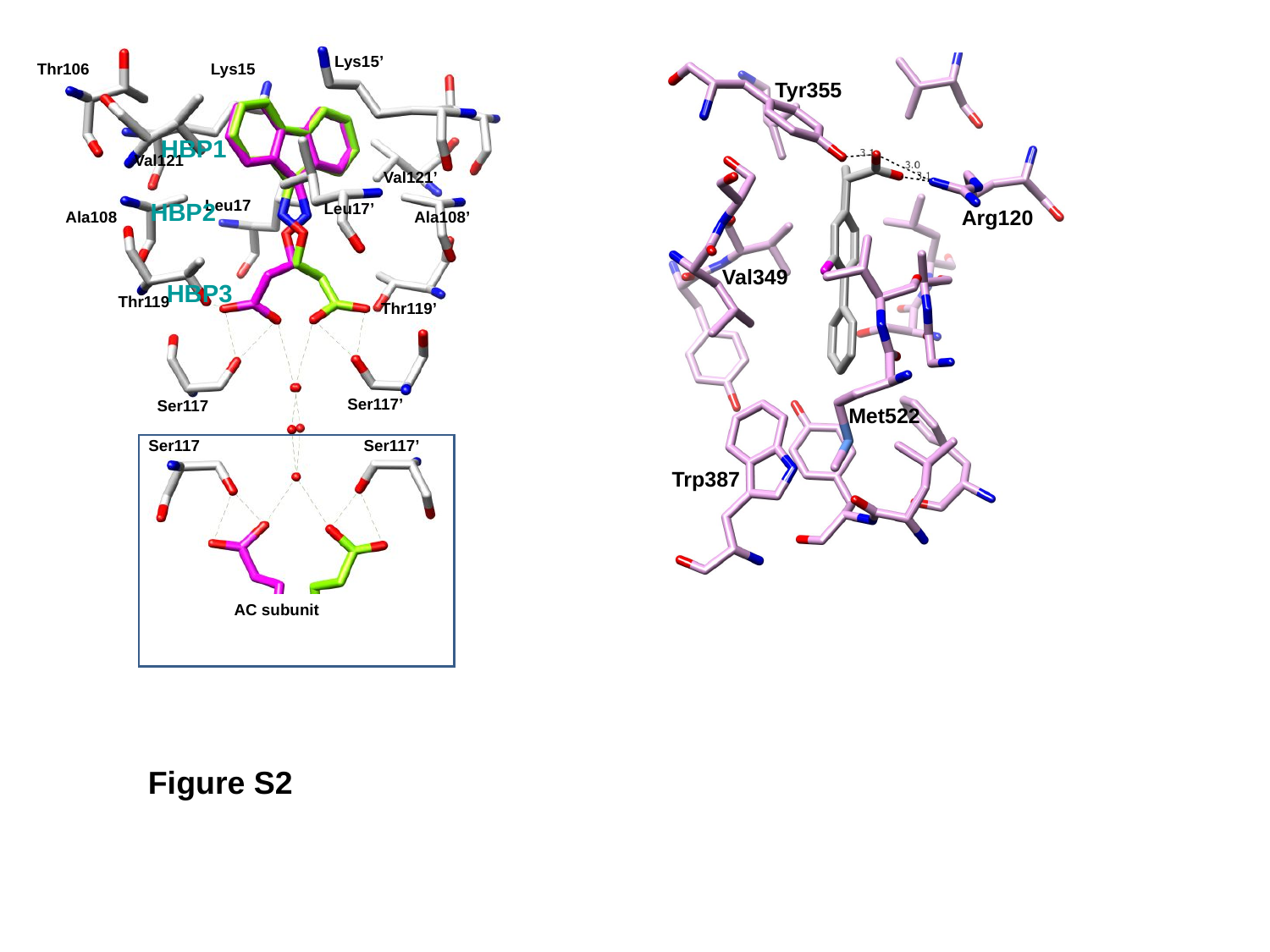

Lys15’
Thr106
Lys15
Val121
Val121’
Leu17
Leu17’
Ala108
Ala108’
Thr119
Thr119’
Ser117’
Ser117
Ser117
Ser117’
AC subunit
Tyr355
HBP1
HBP2
Arg120
Val349
HBP3
Met522
Trp387
Figure S2
